# Loss of tumoral expression of soluble IL-6 receptor is associated with disease progression in colorectal cancer

**DOI:** 10.1038/sj.bjc.6605827

**Published:** 2010-09-07

**Authors:** Y Okugawa, C Miki, Y Toiyama, H Yasuda, T Yokoe, S Saigusa, J Hiro, K Tanaka, Y Inoue, M Kusunoki

**Affiliations:** 1 Division of Reparative Medicine, Department of Gastrointestinal and Pediatric Surgery, Institute of Life Sciences, Mie University Graduate School of Medicine, 2-174 Edobashi, Tsu, Mie 514-8507, Japan

**Keywords:** colorectal cancer, interleukin-6, soluble interleukin-6 receptor, trans-signalling

## Abstract

**Background:**

Interleukin-6 (IL-6) binds both the membrane and soluble forms of the IL-6 receptor (sIL-6R), which induces a complex with gp130, and proliferation of tumour cells. The aim of this study is to clarify the relationship between tumoral sIL-6R expression and disease progression in colorectal cancer patients.

**Methods:**

We measured tissue concentrations of sIL-6R in tumour and normal mucosa from 161 colorectal cancer patients undergoing surgery, and in supernatants from colon cancer cell lines. The expression of IL-6, IL-6R and gp130 was evaluated by immunohistochemical analysis.

**Results:**

Loss of tumour expression of sIL-6R as defined by sIL-6R Ca/N ratio <1.0 was significantly associated with factors reflecting disease progression, and was an independent prognostic factor not only in all the patients in this study, but also in the patients with curative intent. Colon cancer cell lines produced sIL-6R *in vitro*, and the production of sIL-6R in cancer cell lines was stimulated by cytokine stimulation. Immunohistochemistry revealed that loss of tumour expression of sIL-6R was significantly inversely correlated with intense IL-6 expression in the cytoplasm of cancer cells. In addition, tumoral IL-1*β* expression was significantly correlated with sIL-6R expression.

**Conclusion:**

Loss of tumour expression of sIL-6R is associated with colorectal cancer disease progression.

The inflammatory response has been shown to be intimately involved in colorectal cancer progression and correlates with a poor outcome in patients undergoing resection for colorectal cancer. A mechanistic link between inflammation and colorectal cancer progression is supported by the finding that precursor lesions of colorectal cancer often show inflammatory histological features (Higaki *et al*, 1999). In addition, the inflammatory response promotes carcinogenesis by damaging DNA (Jaiswal *et al*, 2000), stimulating angiogenesis and cell proliferation, and inhibiting apoptosis (Jackson *et al*, 1997). These recent reports suggest that inflammation is intimately involved in carcinogenesis and cancer progression in colorectal cancer.

Interleukin-6 (IL-6) is a pleiotropic cytokine that has a key role in the induction and maintenance of the inflammatory response with many ascribed effects, including immunoregulation, angiogenesis, and osteoclast activation (Taga and Kinoshita, 1997; Lauta, 2003; Sohara *et al*, 2005; Hong *et al*, 2007). Several human tumour cell lines are known to produce IL-6 (Kawano *et al*, 1988; Miki *et al*, 1989; Meir *et al*, 1990; Siegall *et al*, 1990; Watson *et al*, 1990; Lee *et al*, 1992; Ohsaki *et al*, 1992; Takizawa *et al*, 1993; Oka *et al*, 1996). Clinically, serum IL-6 levels increase in colorectal cancer patients relative to healthy controls, and is correlated with tumour size, suggesting its potential as a prognostic marker of tumour progression (Galizia *et al*, 2002; Chung and Chang, 2003). Experimentally, IL-6 has been implicated as a promoter of cancer growth by enhancing colony formation of human colon carcinoma cells in a dose-dependent manner *in vitro* (Schneider *et al*, 2000). These data suggest that IL-6 has a pivotal role in the progression of colorectal cancer.

Interleukin-6 exerts its activity on target cells by binding to the ligand-specific receptor IL-6R, which exists aseither a membrane-bound or a soluble form (sIL-6R). Classic signalling of IL-6 involves IL-6 binding to target cells bearing the membrane-associated IL-6R. Alternatively, IL-6 can activate cells lacking the membrane-associated IL-6R when IL-6 binds the naturally occurring sIL-6R in a process called IL-6 trans-signalling (Jones *et al*, 2001), whereby IL-6/sIL-6R complexes activate the gp130 signalling receptor on the cell surface. Soluble forms of the IL-6 receptor is released into the circulation by T cells, macrophages, and granulocytes by proteolytic cleavage from the cell surface (Mullberg *et al*, 1993), or by differential messenger RNA splicing (Horiuchi *et al*, 1994).

Although most soluble receptors function as functional antagonists for their specific cytokine, sIL-6R shows agonistic activity (Rose-John and Heinrich, 1994). Given the inflammatory properties of IL-6, sIL-6R could emerge as an important inflammatory mediator at different stages of colon cancer pathogenesis through IL-6 signalling in colorectal cancer cells. However, no study has yet clarified the relationship between the expression of intra-tumoral sIL-6R and cancer progression in colorectal cancer patients.

In this report we evaluated the relationship between intra-tumoral expression of sIL-6R and disease progression in patients with colorectal cancer. We also examined the significance of IL-6 trans-signalling in the cancer microenvironment with colorectal cancer progression.

## Patients and methods

Among the 243 consecutive patients who underwent surgery for colorectal cancer from January 1996 to January 2003 at Mie University Hospital, Japan, 109 patients who were followed up over a 5-year period for evidence of recurrence, and 52 patients who died of primary or recurrent disease within 5 years of the follow-up period were enrolled in this study. Another factor for inclusion in this study was the retention of excellent cancer and normal tissue samples. Out of the 161 patients, 104 were male. The mean age was 64.6 years (range 37–86 years). No perioperative mortalities were observed among these patients. No patients had received chemotherapy or radiation therapy before surgery.

The location of the tumours and distant metastases were determined by barium enema, colonoscopy, computed tomography (CT), and magnetic resonance imaging (MRI). The primary lesion was located in the rectum in 70 patients, the sigmoid colon in 48 patients, the ascending colon in 30 patients, the transverse colon in 8 patients, and the descending colon in 5 patients. In all, 26 patients were diagnosed with synchronous liver metastasis, and 3 patients with both liver metastasis and peritoneal dissemination. Resection of the primary tumour was carried out in all patients, and simultaneous partial hepatectomy for liver metastasis was carried out in 12 patients. In total, 12 patients had poorly differentiated adenocarcinomas, while 149 patients had well differentiated or moderately differentiated adenocarcinomas. All patients were classified according to the Union Internationale Contre le Cancer (UICC) classification system, based on resected specimens. There were 38 patients with UICC stage I (T1-2N0M0), 36 patients with UICC stage II (T3-4N0M0), 52 patients with UICC stage III (TXN1-2M0), and 35 patients with UICC stage IV (TXNXM1) disease. Stage III and IV patients received fluorouracil-based chemotherapy, whereas stage I and II patients received no postoperative adjuvant therapy. Patients were observed at 3-month intervals for 24 months after completion of surgery, followed by every 6 months for 3 years, and then yearly. History was taken and a physical examination was carried out at each visit, and chest X-ray, colonoscopy, and CT were carried out annually. The median follow-up time was 63.2 months (mean: 59.9±38.7). Among the 161 patients studied, 56 patients died because of primary or recurrent disease. The clinicopathological parameters studied for their possible prognostic value were as follows: T classification, vessel involvement, lymphatic invasion, lymph node metastases, and distant metastasis.

Fresh surgical specimens of primary colorectal carcinoma were taken from the distant stump of the resected specimens under sterile conditions, and were placed immediately in liquid nitrogen and stored at −80°C until assayed.

### Tissue concentrations of sIL-6R and IL-1*β*

In all, 322 specimens (161 cancer and 161 normal colorectal mucosa) were prepared for analysis of tissue expression of sIL-6R and IL-1*β*. These samples were thawed, quickly weighed, and placed in 5 ml of phosphate-buffered saline (PBS). The tissues were homogenised on ice in 1 ml extraction buffer per 100 mg wet weight of tissue using a motor-driven Teflon pestle for 5 min. The tissue extract obtained after centrifugation at 12 000 r.p.m. for 15 min at 4°C was placed in a 200-*μ*l vial, and stored at −80°C. The concentrations of cytokines in these tissues were measured in the supernatant using commercially available enzyme-linked immunosorbent assay (ELISA) kits for both sIL-6R and IL-1*β* (BioSource International, Camarillo, CA, USA). Protein concentrations were measured using a BCA Protein Assay Kit (Pierce Chemical, Rockford, IL, USA). Tissue concentrations were expressed as pg sIL-6R or IL-1*β* per mg total protein. Informed consent was obtained from each subject.

### Immunohistochemical analysis

In total, 52 patients were randomly selected for immunohistochemical analysis. Out of these, 31 patients were male. The mean age was 62.7 years (range 37–81 years). There were 9 patients with UICC stage I, 12 patients with UICC stage II, 16 patients with UICC stage III, and 15 patients with UICC stage IV disease. The median follow-up time was 63.0 months (mean: 59.9±41.0). Out of the 52 patients, 19 patients died because of primary or recurrent disease. Paraffin block specimens were cut into 5-*μ*m sections, and attached to glass slides with melted wax at 65°C. The sections then were dewaxed, hydrated, and incubated in 3% hydrogen peroxide for 30 min. The sections were washed in cold tap water, heated in a microwave oven, and washed three times in PBS (pH 7.4) for 5 min. After washing with PBS, sections were incubated with primary antibodies overnight at 4°C. Non-specific binding was blocked by incubation with blocking solution for 1 h at room temperature. The sections were incubated with appropriate antibody overnight at 4°C: anti-IL-6 (1 : 200; Santa Cruz Biotechnology, Santa Cruz, CA, USA), anti-IL-6R*α* (1 : 200; Santa Cruz Biotechnology), and anti-gp130 (1 : 200; Santa Cruz Biotechnology). The sections were washed and incubated for 30 min at room temperature with an appropriate biotinylated IgG diluted in PBS. The sections were then incubated with avidin/biotin complex (ABC) reagent for 3 h at room temperature. The colour was developed for 90 s using a Vector DAB substrate kit and counterstained with Meyer's haematoxylin (Vector Laboratories, Burlingame, CA, USA). In addition, concentrations of IL-6 in tissue extracts from these groups (52 cancer and 52 normal colorectal mucosa) were measured using a commercially available ELISA kit (BioSource International). Tissue concentrations were expressed as pg IL-6 per mg total protein.

### Evaluation of degree of antibody reactivity

The specificity of the immunoreaction was verified by staining known positive and negative control tissue sections. The degree of anti-IL-6, anti-IL-6R*α*, and anti-gp130 reactivity in each tissue section was scored according to the percentage of stained cancer cells and cancer stromal tissues in the section. In this study, cancer cells and cancer stromal tissues with more than 50% stained cells were defined as ‘positive’, and others (<50%) as ‘reduced’, as described previously (Komatsu *et al*, 2000). The slides were evaluated three times by three independent investigators, who were blinded to the nature of the specimens and antibodies used.

### Cell culture conditions

The human colon cancer cell line HT-29 was obtained from the Cell Resource Center for Biomedical Research, Institute of Development, Aging and Cancer, Tohoku University, Japan. RPMI 1640 medium was purchased from Sigma (St Louis, MO, USA). Fetal bovine serum (FBS) and non-essential amino acids (NEAAs) were purchased from GIBCO-BRL (Grand Island, NY, USA). Recombinant human IL-1*β* was purchased from Peprotech House (London, UK). Recombinant human IL-1 receptor antagonist (IL-1RA) was purchased from R&D Systems Inc. (Minneapolis, MN, USA). All cells were maintained in RPMI 1640 medium containing 10% FBS, and prepared to a final concentration of 1 × 10^5^ cells ml^−1^, and cultured in six-well plates. After 24 h starvation in serum-free medium, serum-free medium with or without IL-1*β* (10 ng ml^−1^) and IL-1RA (100 ng ml^−1^) were added. The conditioned medium (CM) was harvested at 0, 1, 3, 6, 12, and 24 h and stored at −80°C. Supernatant concentrations of sIL-6R and soluble gp130 (sgp130) (Quantikine, Minneapolis, MN, USA) were measured by ELISA.

### Total RNA extraction and semi-quantitative RT–PCR analysis

To clarify that IL-1*β* enhances IL-6R splice variant expression and/or the shedding of the membrane-bound form of IL-6R, we carried out semi-quantitative RT–PCR analysis to detect both IL-6R and sIL-6R, which lack the transmembrane (TM) domain (Horiuchi *et al*, 1994). Cells were maintained in RPMI 1640 medium containing 10% FBS, and seeded at a final concentration of 1 × 10^5^ cells ml^−1^, and cultured in six-well plates. After 24 h starvation in serum-free medium, serum-free medium with or without IL-1*β* (10 ng ml^−1^) and IL-1RA (100 ng ml^−1^) were added. Cells were collected at 0, 6, and 12 h as follows: tumour cells were washed with PBS and harvested with trypsin. Following this, total RNA was extracted using an RNeasy Midi kit (Qiagen Inc., Chatsworth, CA, USA) according to the manufacturer's instructions. Primers used for the PCR of IL-6R were as follows: either IL-6R or sIL-6R primers (sense, 5′-ACGCCTTGGACAGAATCCA-3′, and antisense, 3′-TGGCTCGAGGTATTGTCAGA-5′) as described previously (Horiuchi *et al*, 1994). Primers for *β*-actin were designed with Primer3 software (Biology Workbench Version 3.2, San Diego Supercomputer Center, at the University of California, San Diego, CA, USA). Sequences were as follows: *β*-actin (sense, 5′-ACAGAGCCTCGCCTTTGC-3′, and antisense, 3′-GCGGCGATATCATCATCC-5′). Optimal cycling parameters in the linear phase of amplification consisted of 35 cycles of 1 min denaturation at 91°C, 1 min annealing at 61°C, and 1 min elongation at 72°C for selected genes. Control PCR (35 cycles) was also performed for *β*-actin as a standard for sample normalisation. Amplified products were separated electrophoretically, visualised, and photographed under UV light after ethidium bromide staining, and quantified by CA Analyzer version 2.0 (ATTO Corporation, Tokyo, Japan).

### Statistical analysis

Statistical analysis was carried out using Medcalc 7.2 for Windows (Broekstraat 52, 9030, Mariakerke, Belgium). Results are expressed as mean±s.d. Mann–Whitney *U*-tests were used to evaluate differences between unpaired observations. Contingency tables among the tissue concentration ratio and clinicopathological factors were analysed using Fisher's exact probability test or χ^2^-analysis. The Wilcoxon rank correlation test was conducted for statistical analysis. Actuarial survival curves were obtained using the Kaplan–Meier method, and comparisons were made using log-rank tests. Prognostic factors were examined by univariate and multivariate analyses (Cox proportional hazards model). Two-sided *P*-values <0.05 were considered to be statistically significant.

## Results

### Loss of tumoral sIL-6R expression associates with clinicopathological scores

The concentrations of sIL-6R in cancer tissues ranged from 218.9 to 24 648 pg per mg protein, with a mean level of 1863.5±2474.3 pg per mg protein. The concentrations of sIL-6R in normal mucosa ranged from 108.3 to 19 995 pg per mg protein, with a mean level of 1808.8±2146.1 pg per mg protein. The ratio between the concentrations of sIL-6R in cancer tissue and normal mucosa (sIL-6R Ca/N ratio: cancer tissue sIL-6R concentration divided by normal mucosa sIL-6R concentration) ranged from 0.096 to 6.631 with a mean value of 1.262±1.156. The ROC curve analysis showed that the best cut-off value was 1.021 (sensitivity 73.1%, specificity 56.9%). Thus, the cut-off value was set at 1.0 and was used to classify the tumoral expression of sIL-6 as over-expression or under-expression compared with normal mucosa. Patients with a sIL-6R Ca/N ratio of <1.0 were considered to have lost tumoral expression of sIL-6R (*n*=84), whereas those with values 1.0 were considered to have high tumoral expression of sIL-6R (*n*=77). Loss of tumoral expression was found in 52% of the patients, and in 47% of patients undergoing surgery with curative intent.

**Figure 1:**
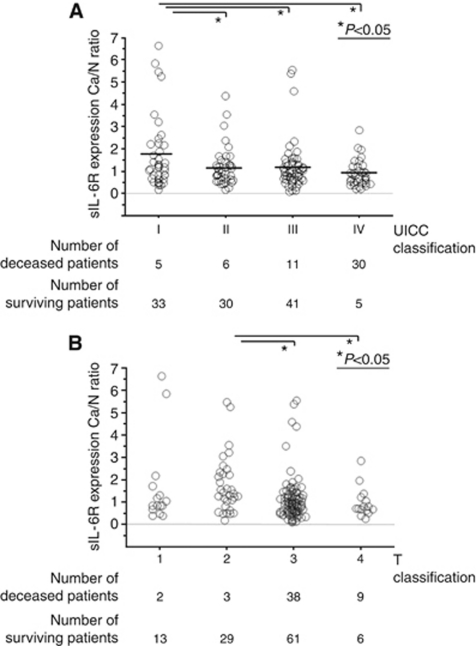
(**A**) Scattergram of the sIL-6R Ca/N expression ratios with UICC classification in 161 colorectal cancer patients. (**B**) Scattergram of the sIL-6R Ca/N expression ratios with T classification in 161 colorectal cancer patients.

The mean level of the sIL-6R Ca/N expression ratios in stage I patients was significantly higher than those in stage II, III or IV patients (Figure 1A). Table 1 shows the relationship between the sIL-6R Ca/N expression ratio and clinicopathological findings. Loss of tumoral sIL-6R expression was associated with factors showing disease progression, such as T classification, the presence of distant metastasis, and the progression of UICC classification. In addition, the mean level of the sIL-6R Ca/N expression ratio in the T2 patients was significantly higher than the levels in T3 or T4 patients (Figure 1B).

### Loss of tumoral sIL-6R expression correlates with poorer prognosis

**Figure 2:**
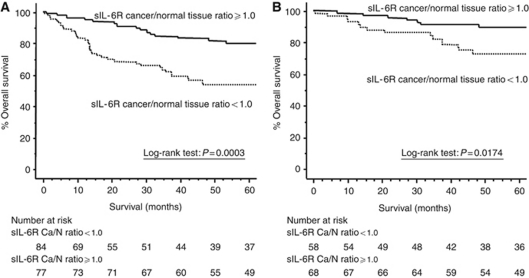
(**A**) Kaplan–Meier data of the actual 5-year survival rates of all patients, according to sIL-6R Ca/N expression ratios. (**B**) Kaplan–Meier data of the actual 5-year survival rates of UICC stage I, II and III patients undergoing potentially curative surgery except synchronous distant metastasis according to sIL-6R Ca/N expression ratios.

Figure 2A shows the actual survival curves for all colorectal carcinoma patients, subdivided by their sIL-6R Ca/N expression ratio. Patients who lost tumoral sIL-6R expression had significantly poorer prognoses than those with higher tumoral sIL-6R expression (log-rank test, *P*=0.0003). Figure 2B shows the actual survival curves for UICC stage I, II, and III patients undergoing potentially curative surgery. The curative intent patients who lost tumoral sIL-6R expression also had significantly poorer prognoses than those with higher tumoral sIL-6R expression (log-rank test, *P*=0.0174). Table 2a reveals the resulting risk ratios and 95% confidence intervals (95% CIs) calculated by Cox proportional hazards analysis in all colorectal cancer patients. In this analysis, hepatic metastasis and loss of tumoral sIL-6R expression were independent risk factors for a poor prognosis. Cox proportional hazards analysis in UICC stage I, II, and III patients similarly showed that loss of tumoral sIL-6R expression was an independent risk factor for a poor prognosis (Table 2b).

### Interleukin-6-expression is correlated with a poorer prognosis for colorectal cancer patients

**Figure 3:**
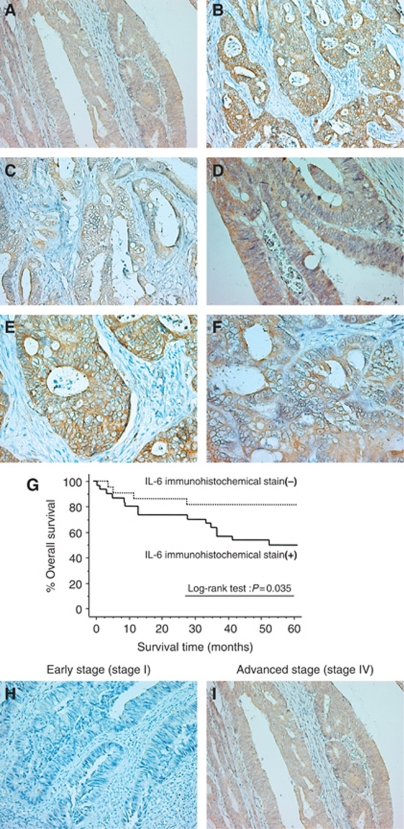
(**A**–**F**) Typical examples of immunohistochemical staining for IL-6 (**A**, **D**), membrane-associated IL-6R (**B**, **E**), and gp130 (**C**, **F**) in colorectal cancer (**A**, original magnification, × 200; **B**, original magnification, × 200; **C**, original magnification, × 200; **D**, original magnification, × 400; **E**, original magnification, × 400; **F**, original magnification, × 400). (**G**) Kaplan–Meier curves of the actual 5-year survival rates of all patients, segregated by high and low IL-6 expression in cancer cells' cytoplasm. (**H**, **I**) Typical examples of immunohistochemical staining of IL-6 at different disease stages in colorectal cancer (**H**, early stage (UICC Stage I), original magnification, × 200; **I**, advanced stage (UICC Stage IV), original magnification, × 200).

Figure 3A–F shows the results of immunohistochemical staining for IL-6, IL-6R, and gp130 in colorectal cancer tissue. Each protein was intensely expressed within the cancer cells rather than in the cancer stroma. Although IL-6 was expressed in the cytoplasm of the cancer cells, IL-6R was expressed in tumour cell membranes. In addition, gp130 was expressed both in the cytoplasm's cancer cell's and on membranes.Interleukin-6R and gp130 expression in colorectal cancer patients did not show a significant correlation with prognosis (data not shown), whereas the patients with positive IL-6 expression in the cytoplasm of cancer cells had significantly poorer prognoses than those with reduced IL-6 expression (Figure 3G; log-rank test, *P*=0.035). Figure 3H and I show typical examples of IL-6 staining at different stages of disease. In addition, sIL-6R Ca/N expression ratios in patients whose cancer cells' cytoplasm were IL-6 immunoreactive were significantly lower than the ratios in patients with reduced cytoplasmic IL-6 immunoreactivity in cancer cells (0.78±0.41 *vs* 1.56±1.21; *P*=0.0015) (Table 3a). Table 3b shows the quantification of IL-6 immunoreactivity by ELISA, and the data showed that IL-6 immunoreactivity in cancer cells reflected increased IL-6 Ca/N ratios. In contrast, there was no significant relationship between sIL-6R Ca/N expression ratios and IL-6R or gp130 immunoreactivity in the tumour cell membrane.

### Soluble form of IL-6R is secreted by colon cancer cell line itself

**Figure 4:**
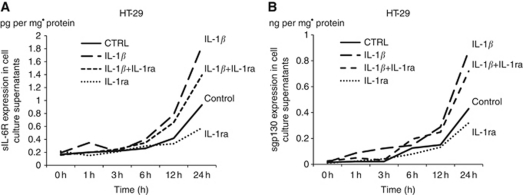
(**A**) The HT-29 colon cancer cells express sIL-6R itself. sIL-6R secretion began 12 h after incubation, and was increased by IL-1*β* stimulation. IL-1RA suppressed the IL-1*β*-induced increase in sIL-6R secretion. (**B**) Although IL-6 expression was initially increased, the expression was gradually downregulated in a time-dependent manner. (**C**) sgp130 expression was increased similar to sIL-6R expression in a time-dependent manner.

In order to clarify whether a loss of sIL-6R in cancer tissue was due to a primary deficiency or because of increased consumption, and to evaluate the complexity of the IL-6 system, we measured sIL-6R and sgp130 protein levels in the supernatants of HT-29 cells at various time points, with or without IL-1*β* and IL-1RA stimulation. Soluble form of IL-6R was secreted from the colon cancer cell line itself, and sIL-6R secretion was upregulated by IL-1*β* stimulation. In addition, IL-1RA suppressed the IL-1*β*-induced enhanced sIL-6R secretion in a time-dependent manner (Figure 4A). The expression of sgp130 also showed the same patterns as those of sIL-6R (Figure 4B).

### The mechanism by which sIL-6R production increases following cytokine stimulation

**Figure 5:**
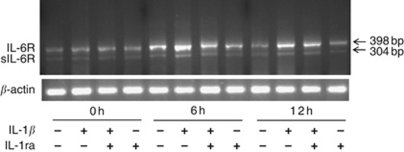
Semi-quantitative RT–PCR analysis to detect both IL-6R (398 bp) and spliced sIL-6R, which lacks the transmembrane domain (304 bp), following cytokine stimulation. IL-1*β* stimulation enhances both IL-6R splice variant expression and the shedding of the membrane-bound form of IL-6R.

We sought to determine whether IL-1*β*-mediated stimulation of sIL-6R was due to increased expression of IL-6R splice variants and/or the shedding of the membrane-bound form of IL-6R. Thus, we used semi-quantitative RT–PCR analysis to detect both IL-6R and spliced sIL-6R, which lacks the TM domain (Horiuchi *et al*, 1994). After 6 and 12 h, IL-1*β* stimulation enhanced both IL-6R splice variant expression and the shedding of the membrane-bound form of IL-6R, compared with those without stimulation (Figure 5). In addition, during IL-1*β* stimulation, IL-1RA inhibited sIL-6R expression by both sIL-6R generation pathways.

### Relationship between tumoral expression of sIL-6R and IL-1*β*

**Figure 6:**
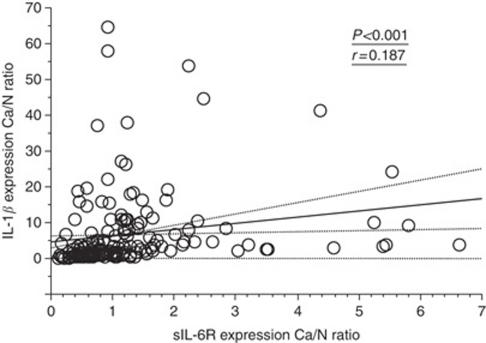
Correlation between the sIL-6R Ca/N expression ratio and the IL-1*β* Ca/N expression ratio. sIL-6R Ca/N expression ratio was positively correlated with the IL-1*β* Ca/N expression ratio.

The concentrations of IL-1*β* in cancer tissues ranged from 0.845 to 757 pg per mg protein, with a mean level of 37.8±80.2 pg per mg protein. The concentrations of IL-1*β* in normal mucosa ranged from 0.38 to 79.4 pg per mg protein, with a mean level of 8.69±10.4 pg per mg protein. The ratio between the concentrations of IL-1*β* in cancer tissue and normal mucosa (IL-1*β* Ca/N ratio: cancer tissue IL-1*β* concentration divided by normal mucosa IL-1*β* concentration) ranged from 0.86 to 116 with a mean value of 6.75±10.8. The tumoral IL-1*β* Ca/N expression ratio was positively correlated with sIL-6R Ca/N expression ratio (Wilcoxon Signed-rank test, *P*<0.001) (Figure 6).

## Discussion

This study is the first report showing a relationship between increased loss of sIL-6R expression in colorectal cancer and disease progression. Many studies have suggested that IL-6 signal transduction is intimately involved in cancer progression (Becker *et al*, 2005; Scheller *et al*, 2006). Several human tumour cell lines have been reported to produce IL-6 (Kawano *et al*, 1988; Miki *et al*, 1989; Meir *et al*, 1990; Siegall *et al*, 1990; Watson *et al*, 1990; Lee *et al*, 1992; Ohsaki *et al*, 1992; Takizawa *et al*, 1993; Oka *et al*, 1996), and an IL-6/IL-6R autocrine loop has been described in several tumours, including oesophageal carcinoma (Oka *et al*, 1996), multiple myeloma (Kawano *et al*, 1988), renal cell carcinoma (Miki *et al*, 1989), and colorectal cancer (Kinoshita *et al*, 1999). Recently, Grivennikov *et al* (2009) showed that the IL-6–Stat3 cascade is an important regulator of the proliferation and survival of tumour-initiating intestinal epithelial cells. Bollrath *et al* (2009) reported that gp130-mediated Stat3 activation has the capacity to regulate IL-6-dependent intestinal epithelial cell survival. It also promotes progression through G1 and G2/M cell cycle phases. Thus, it represents a tumour cell-autonomous mechanism that bridges chronic inflammation to tumour promotion. These reports suggest that IL-6 produced by the tumour cells functions as a growth factor that interacts with specific receptors on the tumour cell surface to induce proliferation or prolongation of survival by the tumour cells.

However, recent reports highlighted the intimate involvement between cancer progression and IL-6 trans-signaling through sIL-6R, rather than the classical IL-6 signalling pathway through IL-6R. Gaillard *et al* (1999) reported that sIL-6R has a high affinity for IL-6, and forms a complex with about 70% of secreted IL-6 in the blood, which enhances the biological effect of released IL-6 (Kovacs, 2001). Becker *et al* (2004) also reported that IL-6 signalling required tumour cell-derived soluble IL-6R rather than IL-6R, and that suppression of TGF-*β*-dependent IL-6 trans-signaling prevented tumour progression *in vivo* .

In this study, increased loss of sIL-6R expression in colorectal cancer was significantly correlated with well-known prognostic factors such as T classification, distant metastasis, and UICC classification. Not only did all of the patients have significantly poorer prognoses when the sIL-6R Cancer/N ratio was less than 1.0, but this effect was also enhanced in patients undergoing surgery with curative intent. These data suggest that increased loss of sIL-6R expression in colorectal cancer is deeply involved in disease progression.

Interestingly, positive IL-6 immunohistochemical reactivity in the cytoplasm of tumour cells was significantly associated with reduced tumoral expression of sIL-6R. Interleukin-6 is synthesised with a signal peptide, which generally leads to co-translational transport into the endoplasmic reticulum which may explain our immunohistochemical results that IL-6 mainly exists in the cytoplasm of cancer cells. As IL-6 produced in cancer cells can freely diffuse across the cell membrane into the stroma (Miki *et al*, 2009), the loss of tumoral sIL-6R expression may reflect either decreased production of sIL-6R itself or increased consumption of sIL-6R by enhanced IL-6/sIL-6R affinity in the cancer stroma. To clarify this hypothesis, we evaluated the kinetics of sIL-6R production from tumour cells using an *in vitro* model.

Using semi-quantitative RT–PCR analysis, we found that IL-1*β* stimulation appeared to increase the production of sIL-6R by both enhanced shedding of the membrane-bound form of IL-6R, and increased expression of the splice variant of IL-6R. Interestingly, we also found that the addition of IL-1RA inhibited sIL-6R production in a time-dependent manner. Interleukin-1RA is an IL-1R antagonist that binds the same receptor on the cell surface as IL-1, preventing IL-1 signalling. In a previous study, we found that IL-1*β* stimulation induced IL-6 production in colorectal cancer cells (Konishi *et al*, 2005), which was counteracted by IL-1RA, suggesting that IL-1RA inhibits the IL-1/IL-6 cascade in colorectal cancer cells. In this study, controlling the IL-1/IL-6 cascade using IL-1RA decreased the production of sIL-6R. In addition, IL-1*β* expression was positively associated with sIL-6R expression. These data suggest that the IL-1–IL-6 cascade in cancer cells has a key role in regulating the kinetics of sIL-6R in an autocrine fashion.

This inverse correlation between IL-6 and sIL-6R has been reported in postoperative changes in serum concentrations under intense surgical stress (Hatada and Miki, 2000; Wakuda *et al*, 2001).Wakuda *et al* (2001) showed the inversely serial change of serum concentration of sIL-6R with IL-6 during the perioperative period, and suggested that sIL-6R level was reduced by consumption in the perioperative period . Recently, Egler *et al* (2008) have shown that serum IL-6 levels were elevated in patients with high-risk neuroblastoma, whereas sIL-6R levels followed an inverse relationship with disease such that lower levels were present in the patients with metastatic disease. These findings suggest that decreased circulating levels of sIL-6R in aggressive malignant disease reflect increased systemic IL-6 affinity for sIL-6R, resulting in systemic consumption of sIL-6R. Interestingly, we also found that the IL-1–IL-6 cascade in cancer cells regulated the kinetics of soluble gp130 (sgp130). Soluble gp130 is the naturally occurring antagonist of the interleukin-6 (IL-6)/sIL-6R complex, and selectively inhibits IL-6 trans-signaling (Narazaki *et al*, 1993). Although we could not determine why sIL-6R detection was reduced by the concomitant enhanced expression of IL-6 in cancer cells' cytoplasm, it may be hypothesised that the increased release of sIL-6R and sgp130 from cancer cells induced increased binding of sIL-6R by both the soluble and membranous forms of gp130 in cancer stroma, resulting in a decrease of sIL-6R in cancer tissue. Obviously, the magnitude of immune cell infiltration might also have an indispensable role in the complex interactions between IL-6 and sIL-6R in cancer stroma, because IL-6, IL-1*β*, and in part sIL-6R are produced by cancer cells, as well as by immune cells. Thus, the increased binding of sIL-6R and the soluble and membranous forms of gp130 arising from immune cells in cancer stroma might also have roles in the decrease of sIL-6R in cancer tissue.

In conclusion, loss of sIL-6R expression in colorectal cancer is significantly correlated with disease progression. The intense correlation between IL-6 expression in cancer cell and a decreased sIL-6R Ca/N ratio may partly reflect exaggerated consumption of sIL-6R by increased IL-6/sIL-6R affinity in the cancer stroma, which maintains a favourable condition for tumour growth. Recently, tocilizumab, a humanised anti-IL-6 receptor antibody, has been used successfully in clinical trials in both adults and children with rheumatological conditions, where IL-6 is known to have a role (Plushner, 2008). In addition, eicosapentaenoic acid, also known as an anti-inflammatory agent, was shown to stabilise the IL-6 response. The therapeutic significance of the blockade of IL-6 production or IL-6 signalling, which includes trans-signaling through the IL-1–IL-6 cascade or sIL-6R, in the cancer microenvironment remains to be clarified in future investigations. Our data suggest that focusing on IL-6 trans-signaling may lead to new therapeutic approaches to control tumour growth in colorectal carcinomas.

## Figures and Tables

**Table 1:** Relationships between cancer/normal tissue ratio of sIL-6R and clinicopathological factors in 161 colorectal patients

		**sIL-6R expression Ca/N ratio**		
**Variable**	***n***	**High⩾1.0 (*n*=77)**	**Low<1.0 (*n*=84)**	***P***
*Gender*				
Male	104	51	53	0.74
Female	57	26	31	
				
*Age (years)*				
<66	77	37	40	>0.99
⩾66	84	40	44	
				
*Pathological T category*				
1	15	7	8	0.0062*
2	32	24	8	
3	99	41	58	
4	15	5	10	
				
*Vessel involvement*				
+	87	46	41	0.21
−	74	31	43	
				
*Lymphatic vessel involvement*				
+	145	70	75	0.80
−	16	7	9	
				
*Lymph node metastasis*				
N0	84	45	39	0.16
N1	77	32	45	
				
*Distant metastasis*				
M0	126	68	58	0.0131*
M1	35	9	26	
				
*UICC stage classification*				
1	38	24	14	0.0129*
2	36	17	19	
3	52	27	25	
4	35	9	26	

Abbreviations: sIL-6R=soluble forms of the interleukin-6 receptor; sIL-6R expression Ca/N ratio=ratio between the concentrations of sIL-6R in cancer tissue and normal mucosa; UICC=Union Internationale Contre le Cancer. ^*^P<0.05.

**Table 2:** Multivariate analysis for predictors of survival in 161 colorectal cancer patients (a) and in UICC stage I, II, and III patients (b)

	**Univariate**			**Multivariate**		
**Variables**	**HR**	**95% CI**	***P*-value**	**HR**	**95% CI**	***P*-value**
*(a)*						
Gender (male *vs* female)	0.76	0.44–1.33	0.61			
Age (<66 *vs* ⩾66)	0.95	0.55–1.64	0.85			
T classification (T1,2 *vs* T3,4)	0.21	0.08–0.52	0.0008*	0.56	0.20–1.55	0.27
Lymphatic vessel involvement (yes *vs* no)	2.08	0.65–6.667	0.22			
Vessel involvement (yes *vs* no)	2.07	1.16–3.70	0.0141*	1.78	0.96–3.31	0.07
Node involvement (yes *vs* no)	2.34	1.33–4.13	0.0032*	1.02	0.54–1.91	0.96
Hepatic metastasis (yes *vs* no)	16.4	8.93–30.3	<0.0001*	12.3	6.25–24.4	<0.0001*
sIL-6R cancer/normal expression ratio (⩾1.0 *vs* <1.0)	0.35	0.19–0.63	0.0006*	0.38	0.20–0.72	0.0032*
						
*(b)*						
Gender (male *vs* female)	1.15	0.36–2.07	0.75			
Age (<66 *vs* ⩾66)	0.86	0.37–1.99	0.73			
T classification (T1,2 *vs* T3,4)	0.47	0.17–1.29	0.14	0.75	0.26–2.11	0.58
Lymphatic vessel involvement (yes *vs* no)	1.44	0.34–6.13	0.62			
Vessel involvement (yes *vs* no)	2.44	0.99–6.02	0.05	2.7	1.07–6.85	0.0362*
Node involvement (yes *vs* no)	1.31	0.57–3004	0.53			
sIL-6R cancer/normal expression ratio (⩾1.0 *vs* <1.0)	0.35	0.14–0.87	0.0230*	0.32	0.13–0.82	0.0169*

Abbreviations: CI=confidence interval; HR=hazard ratio; IL-6=interleukin-6.

^*^P<0.05.

**Table 3a:** Relationship between IL-6, IL-6R and gp130 expressions in cancer cell/stroma and sIL-6R cancer/normal ratio

	***n***	**sIL-6R cancer/normal ratio**	***P-*value**		***n***	**sIL-6R cancer/normal ratio**	***P-*value**
*IL-6 expression in cancer cell*				*IL-6 expression in cancer stroma*			
+	30	0.78±0.41	0.0015*	+	3	0.63±0.47	0.19
−	22	1.56±1.21		−	49	1.14±0.94	
							
*IL-6R expression in cancer cell*				*IL-6R expression in cancer stroma*			
+	13	1.10±0.83	0.68	+	1	1.595	0.22
−	39	1.11±0.96		−	51	1.10±0.93	
							
*gp130 expression in cancer cell*				*gp130 expression in cancer stroma*			
+	6	0.98±0.52	0.93	+	2	0.69±0.13	0.51
−	47	1.12±0.96		−	50	1.13±0.94	

Abbreviations: IL-6=interleukin-6; sIL-6R=soluble form of interleukin-6 receptor. ^*^P<0.05.

**Table 3b:** Relationship between IL-6 expression in cancer cell/stroma and IL-6 cancer/normal ratio

	***n***	**IL-6 cancer/normal ratio**	***P-*value**		***n***	**sIL-6R cancer/normal ratio**	***P-*value**
*IL-6 expression in cancer cell*				*IL-6 expression in cancer stroma*			
+	30	6.72±9.75	0.0288*	+	3	6.28±9.38	0.79
−	22	3.05±4.79		−	49	5.10±8.20	

Abbreviations: IL-6=interleukin-6; sIL-6R=soluble form of interleukin-6 receptor. ^*^P<0.05.
